# Do Socio-Economic Determinants Influence DPYD Testing? A Real-World Study of 1478 Cancer Patients Receiving Fluoropyrimidine Chemotherapy

**DOI:** 10.3390/medsci14010049

**Published:** 2026-01-17

**Authors:** Bahaaeldin Baraka, Navin Mathiyalagan, Maryam Al-Ani, Gaurav Mohindru, Torran Semple, Hrushikesh Divyateja, Grazziela Figueredo, Philip Quinlan, Guruprasad Padur Aithal, Srinivasan Madhusudan

**Affiliations:** 1 Department of Oncology, Nottingham University Hospitals, Nottingham NG5 1PB, UK; bahaaeldin.baraka@nhs.net (B.B.); n.mathiyalagan@nhs.net (N.M.); maryam.alani@nhs.net (M.A.-A.); gauvrav.mohindru1@nhs.net (G.M.); 2 Department of Engineering Science, University of Oxford, Oxford OX1 3PJ, UK; torran.semple@eng.ox.ac.uk; 3 Department of Chemical Pathology, Nottingham University Hospitals, Nottingham NG5 1PB, UK; h.divyateja@nhs.net; 4 Department of Computer Science, University of Nottingham, Nottingham NG8 1BB, UK; g.figueredo@nottingham.ac.uk; 5 Health Informatics, NIHR Nottingham Biomedical Research Centre, Nottingham NG7 2UH, UK; philip.quinlan@nottingham.ac.uk; 6 NIHR Nottingham Biomedical Research Centre, Nottingham University Hospitals NHS Trust, The University of Nottingham, Nottingham NG5 1PB, UK; guru.aithal@nottingham.ac.uk; 7 Biodiscovery Institute, School of Medicine, University of Nottingham, Nottingham NG5 1PB, UK

**Keywords:** *DPYD*, 5-fluorouracil, capecitabine, real-world data

## Abstract

**Background:** The *DPYD* gene encodes dihydropyrimidine dehydrogenase (DPD), an enzyme essential for metabolising chemotherapeutic agents such as capecitabine, 5-fluorouracil (5-FU), and tegafur. Variants in this gene can increase the toxicity of these treatments. **Methods:** This study analysed data from 1478 cancer patients at Nottingham University Hospitals who received chemotherapy between December 2021 and December 2023. The study assessed the prevalence of *DPYD* variants across different tumour types, ethnic groups, and socioeconomic factors. **Results:** Overall, *DPYD* variants were identified in 7% of patients, with higher rates in colorectal cancer (7.9%) and among Caucasian patients (7.4%). The most frequent variant was c.1129-5923C>G (HapB3), found in 75.7% of variant-positive cases. No significant differences in DPYD testing rates were observed across socioeconomic groups or between ethnic backgrounds within our cohort. **Conclusions:** *DPYD* variants were prevalent in 7% of the cohort, and testing access was not influenced by socioeconomic status.

## 1. Introduction

Dihydropyrimidine dehydrogenase (DPD) is a key enzyme that metabolises fluoropyrimidine-based chemotherapy drugs, including 5-fluorouracil (5-FU), capecitabine, and tegafur [1]. Genetic changes in the *DPYD* gene can reduce or eliminate DPD activity, which may result in severe and potentially life-threatening toxicity during treatment [2]. As a result, *DPYD* testing has become increasingly important in clinical practice, supported by major regulatory bodies such as the European Medicines Agency (EMA) and the Medicines and Healthcare products Regulatory Agency (MHRA) [3].

NHS England and NHS Improvement have also issued an urgent clinical policy requiring *DPYD* screening before initiating fluoropyrimidine treatment [4]. This policy specifies testing for four key *DPYD* variants that are known to significantly increase the risk of fluoropyrimidine-related toxicity, including severe diarrhoea, blood-related side effects, and mucositis [5]. The guideline further states that this test needs to be performed only once at the start of treatment, as the results will remain valid for all subsequent treatments with fluoropyrimidine-based medications. The UK Chemotherapy Board is also expected to guide appropriate dose adjustments for patients with *DPYD* variants [6]. Fluoropyrimidine medications, such as 5-fluorouracil (5-FU) and capecitabine, remain essential treatments for many solid tumours, including breast, colorectal, and pancreatic cancers. However, 20–40% of patients experience significant treatment-related toxicity, often due to reduced activity of the dihydropyrimidine dehydrogenase (DPD) enzyme [4,5]. Germline variations in the *DPYD* gene, such as c.1905+1G>A (DPYD*2A), c.1679T>G, and c.2846A>T, are well known to decrease DPD function and increase the risk of severe toxicity [7,8]. Routine *DPYD* testing can help avoid significant side effects and make treatment safer [6]. Testing for tier-1 variations, which have an allele frequency of at least 0.1% and are closely linked to fluoropyrimidine toxicity, is what current lab standards require. An example of a tier-1 variant is c.1905+1G>A (*DPYD*) [9].

Despite these recommendations, the actual frequency of *DPYD* variants across different ethnic groups and socioeconomic backgrounds is still not well understood. Furthermore, the extent to which socioeconomic disadvantage affects access to *DPYD* testing remains unclear. This study seeks to address this deficiency by examining data from 1478 cancer patients undergoing fluoropyrimidine chemotherapy at Nottingham University Hospitals, representing a heterogeneous ethnic and socio-economic demographic.

## 2. Materials and Methods

### 2.1. Patients

This retrospective analysis included 1478 cancer patients who received fluoropyrimidine-based chemotherapy (5-FU, capecitabine, or trifluridine/tipiracil) at Nottingham University Hospitals between December 2021 and December 2023. The study received approval from the Clinical Effectiveness Team (Reference: 23-739C, approval date: 15 December 2023). The study was considered an audit under NHS research governance. All data were anonymised, and patient confidentiality was maintained in accordance with institutional data protection and research governance guidelines. All patients provided informed consent for chemotherapy treatment. Individual patient consent was not required for this retrospective audit. Data collection included demographic information, tumour types, and DPYD test results for all participants. *DPYD* genotyping was conducted using assays that targeted the four most common variants: c.1129-5923C>G, c.1905+1G>A, c.2846A>T, and c.1679T>G. Patients were eligible for inclusion if they had been diagnosed with cancer and treated with fluoropyrimidine-based chemotherapy at Nottingham University Hospitals during the specified period. All included patients also underwent DPYD genotyping for the common variants, and demographic data, tumour type information, and postcodes were available for geocoding purposes. The postcodes were linked to Lower-layer Super Output Area (LSOA), a geographical grouping of 1000 to 3000 people. The LSOA was linked to the 2019 data from the Office of National Statistics. Patients were excluded if DPYD test results were unavailable, if key demographic or clinical data were incomplete or missing, or if they were not treated with fluoropyrimidine-based chemotherapy or if LSOA or deprivation data was not available for the supplied postcode.

### 2.2. Statistical Analysis

The study was designed as a retrospective analysis of 1478 cancer patients who received fluoropyrimidine-based chemotherapy and underwent DPYD testing. Data were analysed using IBM SPSS 28.0 (Armonk, NY, USA: IBM Corp.). Descriptive statistics were used to summarise demographic variables, tumour types, and the prevalence of DPYD variants. The overall prevalence of DPYD variants was calculated with 95% confidence intervals. Bivariate analysis was conducted to examine the relationship between socioeconomic status (measured by the Index of Multiple Deprivation, IMD) and *DPYD* testing rates [10]. Choropleth maps were generated to visualise the spatial distribution of *DPYD* testing rates relative to deprivation indices, using geocoded patient addresses and LSOAs.

To distinguish between ‘deprived’ and ‘affluent’ areas, we utilised the Index of Multiple Deprivation (IMD). LSOAs in deciles 1–5 (representing the most deprived 50% of areas nationally) were categorised as deprived, while those in deciles 6–10 were categorised as affluent. Within our sample, 155 LSOAs were classified as deprived and 27 as affluent; this distribution reflects Nottingham’s status as a city with higher-than-average levels of deprivation relative to other English districts.

Statistical significance was defined as *p* < 0.05. Mann–Whitney U tests were used to assess differences in *DPYD* testing rates between the most and least deprived LSOAs. No significant variation was found between these groups (*p*-value = 0.74).

Maps were generated using QGIS, a geographic information system (GIS) software (version QGIS 3.40.14) that facilitates the visualisation of geocoded data. Here, bivariate choropleth maps are employed to represent two key variables: (i) the DPYD test rate in Nottingham and (ii) levels of deprivation as measured by the Index of Multiple Deprivation (IMD). Both variables are mapped at the level of LSOAs, which are valuable geographical units due to their consistent population densities, typically ranging from 400 to 1200 households, with resident populations between 1000 and 3000 people [7]. The use of LSOAs provides fine-grained analysis, enabling examination of regional variations.

The first variable, *DPYD* test rate, reflects the number of recorded *DPYD* tests per LSOA, adjusted for the number of households in that area. This adjustment is necessary because the number of households in Nottingham’s LSOAs varies from 416 to 1148, with a median of 670. Without this adjustment, apparent differences in *DPYD* test frequency might simply reflect differences in population size across LSOAs. The *DPYD* test rate for any given LSOA can be calculated using the formula:

DPYD test rate (LSOAx) = (DPYD testsₗₛₒₐₓ/householdsₗₛₒₐₓ) × 100

**Figure 1:**
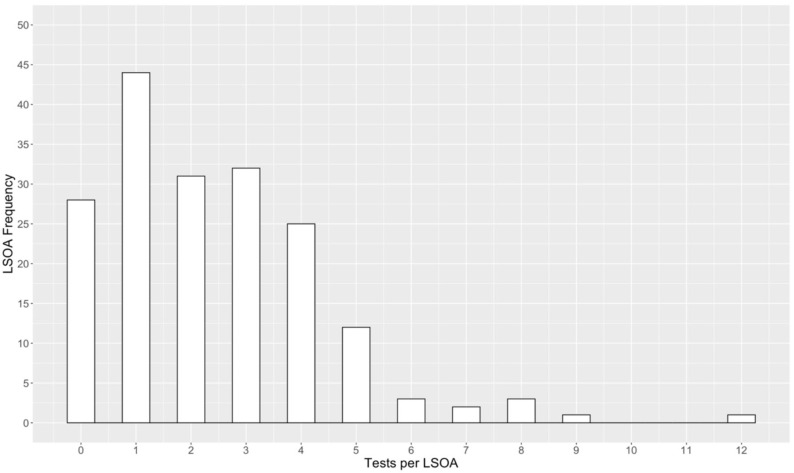
Histogram showing the distribution of DPYD tests per LSOA in Nottingham (n = 182).

**Figure 2:**
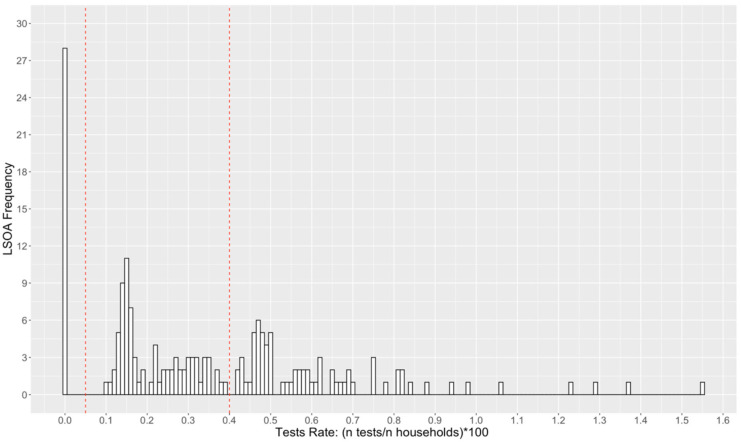
Histogram showing the distribution of DPYD test rate per LSOA in Nottingham (n = 182).

Figure 1 presents the raw DPYD test frequency for each Nottingham LSOA, whereas Figure 2 illustrates the DPYD test rate after adjusting for the population size of each LSOA. Figure 1 shows that the majority of Nottingham’s 182 LSOAs have between 0 and 5 households that have undergone testing. Although the interpretation of Figure 2 is more complex, the *DPYD* test rate provides a more accurate depiction of the proportion of households tested within each LSOA. The red dashed vertical lines in Figure 2 indicate the aggregated categories for *DPYD* test rates, corresponding to levels A1, A2, and A3 on the test rate scale, as used in the bivariate maps.

The second variable of interest, the Index of Multiple Deprivation (IMD), is calculated for every LSOA across the UK. IMD measures various forms of deprivation and is weighted by several factors: income (22.5%), employment (22.5%), health deprivation and disability (13.5%), education, skills, and training (13.5%), crime (9.3%), barriers to housing and services (9.3%), and living environment (9.3%) (https://www.gov.uk/government/collections/english-indices-of-deprivation, accessed 1 May 2024). The primary purpose of IMD is to rank LSOAs from the least deprived to the most deprived. Since this ranking is done on a national scale, many LSOAs in economically disadvantaged cities like Nottingham rank as more deprived than the national average. To account for this, two versions of the *DPYD* versus IMD bivariate maps are used. The first version utilises an IMD variable based solely on Nottingham’s LSOAs (i.e., relative to the median IMD rank within Nottingham). In contrast, the second version uses a national comparison (i.e., relative to the median LSOA rank in England). The IMD indices for Nottingham (IMDN) and England (IMDE) are outlined below:

IMD_n_ = (LSOA rank in Nottingham)/(Nottingham median IMD rank)

IMD_e_ = (LSOA rank in Nottingham)/(English median IMD rank)

**Figure 3:**
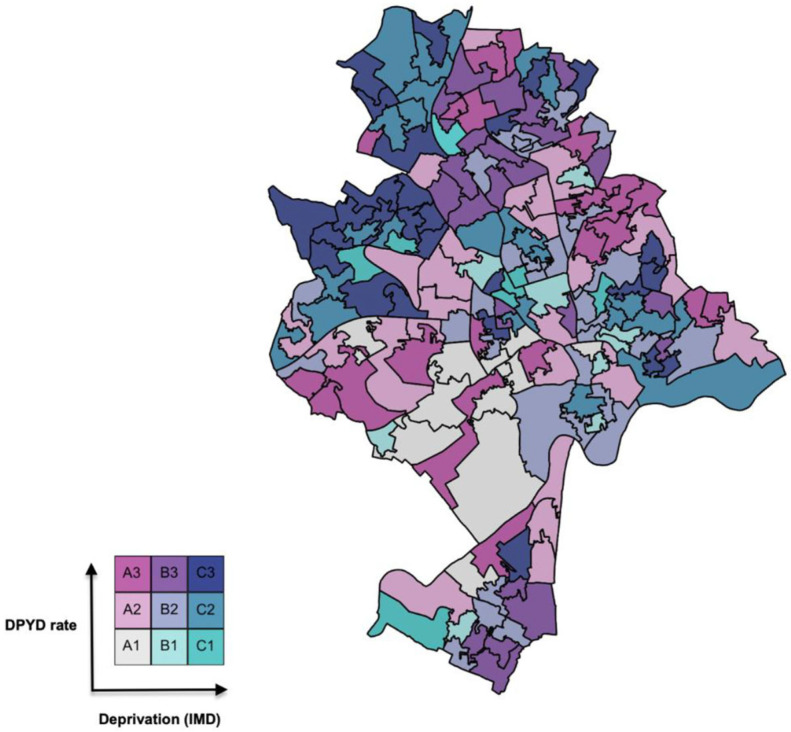
Deprivation (Nottingham baseline, i.e., IMDN) versus DPYD test rate in Nottingham.

**Figure 4:**
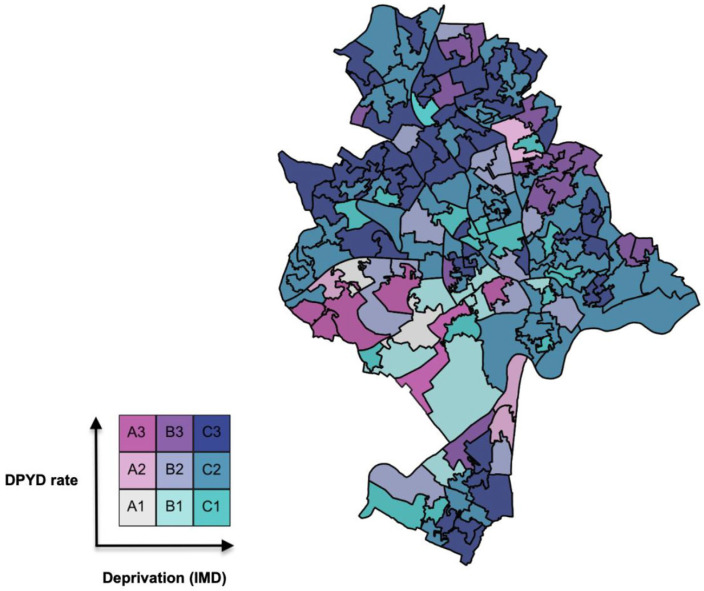
Deprivation (English baseline, i.e., IMDE) versus DPYD test rate in Nottingham.

Figure 3 and Figure 4 display the bivariate maps of DPYD test rate versus IMD in Nottingham LSOAs for the Nottingham and English IMD baseline, respectively. Table 1 provides the numerical interpretation of the legend in each figure.

## 3. Results

A total of 1478 cancer patients treated with fluoropyrimidine-based chemotherapy between December 2021 and December 2023 at Nottingham University Hospitals were included in the study (Table 1). The mean age of the patients was 63 years, with a median age of 64 years. Of these patients, 46.7% (n = 690) were male and 53.3% (n = 788) were female.

**Figure 5:**
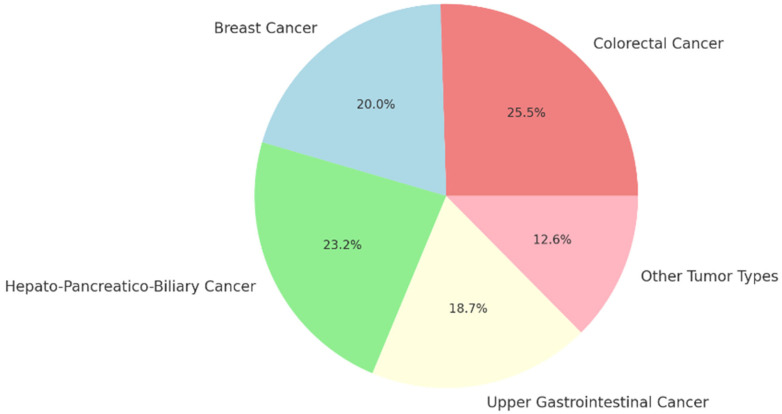
Prevalence of DPYD variants by tumour type.

The overall prevalence of DPYD variants was 7.0% (103/1478; 95% CI: 5.6–8.3%) (Figure 5). The distribution of DPYD variants varied across different tumour types. Among patients with colorectal cancer, the prevalence was 7.9% (56/711; 95% CI: 5.90–9.86%). In patients with breast cancer, the prevalence was 6.2% (20/325; 95% CI: 3.54–8.77%). The prevalence of hepato-pancreatico-biliary cancers was 7.2% (14/194; 95% CI: 3.58–10.86%), and for upper gastrointestinal cancers, it was 5.8% (10/171; 95% CI: 2.33–9.36%). Other tumour types, such as head and neck cancers and neuroendocrine tumours (NET), showed a prevalence of 3.9% (3/77; 95% CI: −0.43–8.22%).

**Figure 6:**
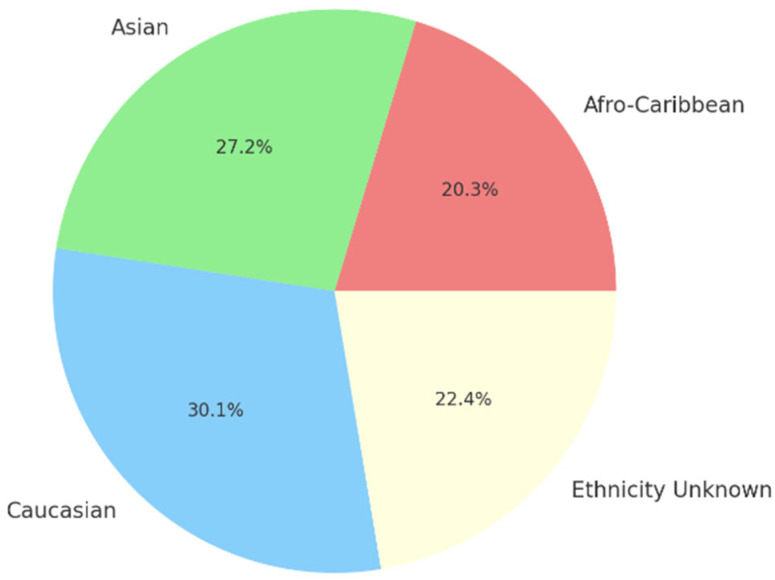
Prevalence of DPYD variants by ethnic groups.

DPYD variants were also examined in different ethnic groups (Figure 6). In Afro-Caribbean patients, the prevalence was 5.0% (1/20; 95% CI: −4.55–14.55%). Among Asian patients, the prevalence was 6.7% (3/45; 95% CI: −0.62–13.95%), while in Caucasian patients, it was 7.4% (89/1207; 95% CI: 5.90–8.85%). In patients with unknown ethnicity, the prevalence of DPYD variants was 5.5% (10/182; 95% CI: 2.18–8.81%).

**Figure 7:**
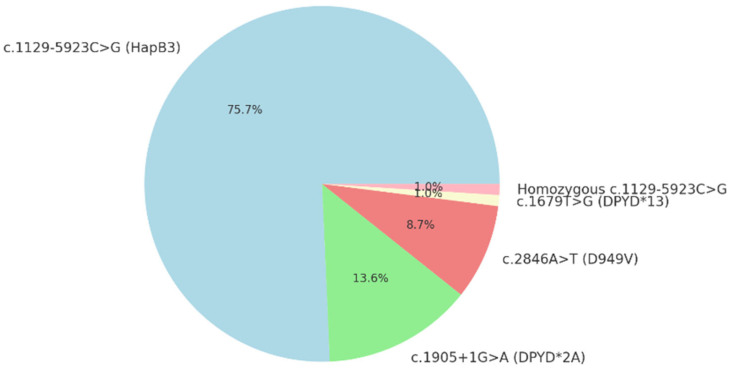
Distribution of DPYD variants identified.

The most common variant observed was c.1129-5923C>G (HapB3), present in 75.7% of variant-positive patients (78/103; 95% CI: 67.0–83.5%). Other identified variants included c.1905+1G>A (DPYD2A), which was present in 13.6% of variant-positive patients (14/103; 95% CI: 7.8–20.4%), and c.2846A>T (D949V), found in 8.7% (9/103; 95% CI: 3.9–14.6%). The c.1679T>G (DPYD13) variant was identified in 1.0% of patients (1/103; 95% CI: 0–2.9%). Additionally, homozygous c.1129-5923C>G was observed in 1.0% of patients (1/103; 95% CI: 0–2.9%) (Figure 7).

The median DPYD test rate in affluent LSOAs was 0.34%, compared to 0.29% in deprived areas. While the raw median suggests a slight increase in affluent areas, the statistical variance test confirms there is no statistically significant evidence to suggest that DPYD test rates differ between these two groups. This suggests that, within this sample, deprivation level was not a primary driver of testing frequency. Patients from both the most deprived and least deprived LSOAs in Nottingham were equally likely to have received DPYD testing. The Mann–Whitney U test indicated no significant variation in testing rates between the most and least deprived areas (*p*-value = 0.74), providing insufficient evidence to reject the null hypothesis that deprivation does not impact DPYD testing accessibility.

To help interpret the bivariate legend in Figure 6 and Figure 7 and the ranks in Table 2, consider cell A3 (dark pink) in Figure 6: LSOAs of this colour have a relatively high DPYD test rate but are in the most deprived third of Nottingham’s LSOAs. The interpretation is the same for Figure 7, except that the deprivation variable is scaled to the English IMD baseline. Cell C1 LSOAs (turquoise) are potentially interesting cases, as these areas suffer relatively high deprivation but have relatively low DPYD testing rates. Cell A3 describes LSOAs with a high DPYD test rate but that are in the most deprived third of all LSOAs in England. As a result, Figure 7 shows many blue LSOAs, as Nottingham is a poorer-than-average city, as discussed previously.

## 4. Discussion

Pharmacogenomics has the potential to improve health outcomes both at the individual and population levels [11,12]. Hence, effective application of pharmacogenomics can improve health equity and medication experience across people from diverse ethnic and socioeconomic backgrounds [11]. Most recent studies have described racial and ethnic disparities with lower rates of germline genetic testing after the cancer diagnosis among Asian, Black, and Hispanic patients compared with White patients in the USA [13]. In addition, racial/ethnic groups underrepresented in genomic studies are also less likely to have access to testing, as they are less likely to have insurance coverage and more likely to be of low-income status [13]. In the current single-centre study, *DPYD* variants were prevalent in 7% of the cohort, with colorectal cancer patients and Caucasians showing the highest rates. Importantly, testing access was not influenced by socioeconomic status. 

*DPYD* is a highly polymorphic gene, with over 1600 known variants, but many are infrequent. Nonetheless, these infrequent mutations can collectively impact a substantial number of individuals. Moreover, dependence solely on the most frequently examined *DPYD* variants may overlook potentially detrimental variants in a significant number of patients. Consequently, the prospective application of Because of these limitations, using full gene sequencing rather than small genotyping panels may provide a more complete picture of a patient’s *DPYD* status. However, this approach also has challenges. Many rare genetic changes are difficult to interpret and are often reported as variants of uncertain significance (VUS), especially since current guidelines are not always designed for pharmacogenomic variants [14,15].

The HapB3 haplotype, which includes the c.1129-5923C>G variant, is particularly important because it helps identify patients with reduced DPD activity. Some laboratories have assumed that the c.1129-5923C>G (HapB3) variant always occurs together with the c.1236G>A variant and therefore use c.1236G>A as an easier substitute when testing for HapB3. Although this simplifies testing, newer studies show that the two variants do not always co-occur across populations. Because of this, using c.1236G>A as a stand-in can lead to incorrect results and may cause some patients at real risk of fluoropyrimidine toxicity to be missed.

These findings reinforce the importance of accurate and comprehensive variant testing, particularly in clinical settings where treatment decisions depend on precise pharmacogenetic information. Direct genotyping of the functional variant should therefore be prioritised, especially for patients who experience unexpected or severe toxicities. Moreover, understanding the population-specific frequency of these variants is critical, as the degree of linkage disequilibrium and allele prevalence can vary substantially between ethnic groups. Including this genetic diversity in testing strategies can make *DPYD* screening both more accurate and fairer across all patient groups [13,16]. In addition to screening for well-known *DPYD* variants, there is growing interest in incorporating *DPYD* testing into routine tumour sequencing, particularly for patients receiving fluoropyrimidine-based chemotherapy [13,14].

As tumour sequencing becomes increasingly prevalent in daily oncology practice, it may provide an excellent opportunity to identify patients at risk of harm [13,15]. Nonetheless, confirming any detected alterations at the germline level is essential to determine their clinical relevance, as somatic mutations within tumour tissue may not accurately reflect a patient’s overall risk of DPD deficiency [13,16,17].

As laboratories adopt next-generation sequencing, their ability to detect rare copy number variants (CNVs) in the *DPYD* gene will improve, helping to explain cases of DPD deficiency or 5-FU toxicity [9]. The prevalence of these CNVs may change across various populations, but their detection could enhance the strategy for personalised chemotherapy regimens. Notwithstanding this potential, the current lack of standardisation for CNV testing in DPYD pharmaceutical genome sequencing precludes its routine recommendation [9].

Patient advocacy groups have played an important role in promoting broader access to *DPYD* testing, raising awareness of its clinical value and encouraging its wider adoption across healthcare systems [18]. This testing may also become an important part of genetic testing for other treatment decisions. As testing methods continue to evolve, laboratories need to adhere to strict quality standards and comply with new guidelines to ensure results remain accurate and useful in clinical practice [14,18,19].

Although *DPYD* testing is essential for reducing chemotherapy-related side effects, more research and improved testing methods are still needed to make sure we can detect all the variants that truly matter for patients’ safety. Incorporating testing for both rare and common variations, as well as novel methodologies such as CNV analysis, will enhance the clinical treatment of patients undergoing fluoropyrimidine-based chemotherapy [14,18,19].

However, our study has several limitations. Its retrospective design restricts the ability to draw causal inferences. Although the sample size was substantial, some ethnic groups were underrepresented, which may limit the generalisability of the results. The assessment of socioeconomic deprivation was confined to a single geographical region, and the indicators used may not fully capture all aspects of healthcare access. Finally, despite adjusting for population size using LSOAs, the possibility of residual confounding cannot be entirely excluded. Nevertheless, since the implementation of mandatory *DPYD* testing in the NHS for all patients requiring fluoropyrimidine-based chemotherapy, our study does suggest equal access is available to all patients.

## 5. Conclusions

In conclusion, *DPYD* variants were found in 7% of patients treated with fluoropyrimidine-based chemotherapy at our centre, with colorectal cancer patients showing the highest prevalence. Our results suggest that social deprivation does not appear to affect access to *DPYD* testing, although this may not apply to other regions or populations. Continued efforts to improve test implementation, such as by increasing awareness, targeted screening for high-risk groups, integrating pharmacogenomics into routine workflows, or enhancing patient education, should be undertaken. Importantly, availability of *DPYD* testing across cancer types will help reduce treatment-related toxicity and improve patient safety.

## Figures and Tables

**Table 1:** Characteristics of Patients and DPYD Variants Distribution.

Characteristic	Data
Age	
Mean (years)	62.69
Median (years)	64
Range (years)	20–89
Sex—no. (%)	
Male	690 (46.7%)
Female	788 (53.3%)
Tumour Site—no. (%)	
Colorectal Cancer	56/711 (7.9%; 95% CI: 5.90–9.86%)
Breast Cancer	20/325 (6.2%; 95% CI: 3.54–8.77%)
Hepato-pancreatico-biliary Cancer	14/194 (7.2%; 95% CI: 3.58–10.86%)
Upper Gastrointestinal Cancer	10/171 (5.8%; 95% CI: 2.33–9.36%)
Other Tumour Types (Head and Neck, NET)	3/77 (3.9%; 95% CI: −0.43–8.22%)
Ethnicity—no. (%)	
Afro-Caribbean	1/20 (5.0%; 95% CI: −4.55–14.55%)
Asian	3/45 (6.7%; 95% CI: −0.62–13.95%)
Caucasian	89/1,207 (7.4%; 95% CI: 5.90–8.85%)
Ethnicity Unknown	10/182 (5.5%; 95% CI: 2.18–8.81%)
DPYD Variants Identified—no. (%)	
c.1129-5923C>G (HapB3)	78/103 (75.7%; 95% CI: 67.0–83.5%)
c.1905+1G>A (DPYD*2A)	14/103 (13.6%; 95% CI: 7.8–20.4%)
c.2846A>T (D949V)	9/103 (8.7%; 95% CI: 3.9–14.6%)
c.1679T>G (DPYD*13)	1/103 (1.0%; 95% CI: 0–2.9%)
Homozygous c.1129-5923C>G	1/103 (1.0%; 95% CI: 0–2.9%)

**Table 2:** Corresponding A, B and C legend codes for DPYD test rate and IMD Legend code.

	DPYD Test Rate	IMD Index
A1	0.00–0.00	0.00–0.67
A2	0.05–0.40	0.00–0.67
A3	0.41–1.60	0.00–0.67
B1	0.00–0.00	>0.67 and <1.34
B2	0.05–0.40	>0.67 and <1.34
B3	0.41–1.60	>0.67 and <1.34
C1	0.00–0.00	1.34–2.00
C2	0.05–0.40	1.34–2.00
C3	0.41–1.60	1.34–2.00

## Data Availability

The original contributions presented in this study are included in the article. Further inquiries can be directed to the corresponding author.

## Abbreviations

The following abbreviations are used in this manuscript:

DPYD	Dihydropyrimidine Dehydrogenase
DPD	Dihydropyrimidine Dehydrogenase Enzyme
5-FU	5-Fluorouracil
IMD	Index of Multiple Deprivation
LSOA	Lower Super Output Area
EMA	European Medicines Agency
MHRA	Medicines and Healthcare products Regulatory Agency
NHS	National Health Service
NET	Neuroendocrine Tumours
IMDN	Index of Multiple Deprivation Nottingham
IMDE	Index of Multiple Deprivation England
SPSS	Statistical Package for the Social Sciences
ESMO	European Society for Medical Oncology
MAP	Molecular Analysis for Precision Oncology
NIHR	National Institute for Health Research
